# The Bradykinin-BDKRB1 Axis Regulates *Aquaporin 4* Gene Expression and Consequential Migration and Invasion of Malignant Glioblastoma Cells via a Ca^2+^-MEK1-ERK1/2-NF-κB Mechanism

**DOI:** 10.3390/cancers12030667

**Published:** 2020-03-13

**Authors:** Ding-Ping Sun, Yuan-Wen Lee, Jui-Tai Chen, Yung-Wei Lin, Ruei-Ming Chen

**Affiliations:** 1 Department of Surgery, Chi Mei Medical Center, Tainan 710, Taiwan; 950232@mail.chimei.org.tw; 2 Cell Physiology and Molecular Image Research Center, Wan-Fang Hospital, Taipei Medical University, Taipei 110, Taiwan; yungweilin@tmu.edu.tw; 3 Department of Anesthesiology, School of Medicine, College of Medicine, Taipei Medical University, Taipei 110, Taiwan; m102093020@tmu.edu.tw (Y.-W.L.); cjuitai@tmu.edu.tw (J.-T.C.); 4 Anesthesiology and Health Policy Research Center, Taipei Medical University Hospital, Taipei 110, Taiwan; 5 Department of Anesthesiology, Shuang Ho Hospital, Taipei Medical University, New Taipei City 235, Taiwan; 6 Graduate Institute of Medical Sciences, College of Medicine, Taipei Medical University, Taipei 110, Taiwan; 7 TMU Research Center of Cancer Translational Medicine, Taipei 110, Taiwan

**Keywords:** glioblastoma, bradykinin-BDKRB1 axis, calcium influx, MEK1-ERK1/2-NF-κB, aquaporin 4, cell migration/invasion

## Abstract

Glioblastoma multiforme (GBM) is the most common form of brain tumor and is very aggressive. Rapid migration and invasion of glioblastoma cells are two typical features driving malignance of GBM. Bradykinin functionally prompts calcium influx via activation of bradykinin receptor B1/B2 (BDKRB1/2). In this study, we evaluated the roles of bradykinin in migration and invasion of glioblastoma cells and the possible mechanisms. Expressions of aquaporin 4 (AQP4) mRNA and protein were upregulated in human glioblastomas. Furthermore, exposure of human U87 MG glioblastoma cells to bradykinin specifically increased levels of BDKRB1. Successively, bradykinin stimulated influx of calcium, phosphorylation of MEK1 and extracellular signal-regulated kinase (ERK)1/2, translocation and transactivation of nuclear factor-kappaB (NF-κB), and expressions of AQP4 mRNA and protein. Concomitantly, migration and invasion of human glioblastoma cells were elevated by bradykinin. Knocking-down BDKRB1 concurrently decreased AQP4 mRNA expression and cell migration and invasion. The bradykinin-induced effects were further confirmed in murine GL261 glioblastoma cells. Therefore, bradykinin can induce AQP4 expression and subsequent migration and invasion through BDKRB1-mediated calcium influx and subsequent activation of a MEK1-ERK1/2-NF-κB pathway. The bradykinin-BDKRB1 axis and AQP4 could be precise targets for treating GBM patients.

## 1. Introduction

According to the World Health Organization (WHO) classification of tumors of the central nervous system (CNS) in 2016, glioblastoma multiform (GBM) is a class IV neoplasm, predominantly arising from transformation of astrocytes [1]. GBM is the most common primary brain tumor, accounting for 16% of brain tumors in the United States [2]. The recommended standard therapy for newly diagnosed GBM patients is surgical resection followed by concurrent adjuvant radiotherapy in combination with chemotherapy [3]. Temozolomide (TMZ), a DNA-alkylating agent, is the first-line chemotherapeutic drug for GBM, but most patients may develop TMZ resistance [4,5]. Hence, the 5-year survival rate of GBM patients is <5%, and the median overall survival time of patients is about 12 months [5,6]. The poor outcomes are due to unique characteristics of glioblastoma cells, including rapid proliferation, resistance to apoptosis, and speedy migration to and invasion of surrounding healthy brain tissues [7]. As a result, uncovering the mechanisms elucidating these exclusive features of glioblastoma cells would certainly be beneficial in establishing de novo strategies for treating GBM.

The kallikrein-kinin system (KKS), consisting of low- and high-molecular weight kininogens, small polypeptides, and a group of enzymes, is involved in inflammatory responses and affects several pleiotropic functions, such as vascular permeability, blood coagulation, and thrombosis [8,9]. Furthermore, KKS components were also identified in the nervous system and work in regulating the neurovascular integrity and progression of neurological diseases [10]. Bradykinin, the most prominent high-molecular weight kininogen produced by the KKS system, can regulate water and ion balance in the body [11]. Regarded as an inflammatory mediator, bradykinin causes blood vessels to dilate and consequently leads to a fall in the blood pressure [12]. In GBM, bradykinin is upregulated and accumulates in tumor sites under pathophysiological conditions such as inflammation and hypoxia, which are correlated with tumor progression [13,14]. Subsequently, the accumulated bradykinin induces additional inflammatory reactions and stimulates the proliferation, migration, and invasion of glioblastoma cells [15,16,17]. Fascinatingly, bradykinin can bind and activate bradykinin receptor B1 and B2 (BDKRB1/2) to regulate the permeability of the blood-tumor barrier in glioblastomas [18,19]. Compared to constitutive expression of BDKRB2, BDKRB1 is an inducible type. Thus, bradykinin may play a crucial role in the progression of brain tumors.

Aquaporin-4 (AQP4), one of the aquaporin water-channel family proteins, is in charge of transporting water through plasma membranes into the cytoplasm [20]. In brain tumors, AQP4 is massively expressed and plays an important role in glioblastoma-related edema [21]. When knocking-down AQP4 expression, glioblastoma cells instantaneously undergo apoptosis [22]. Accordingly, AQP4 participates in maintenance and modulation of activities and functions of glioblastoma cells in brain tumors. Watkins et al. used a mouse model to demonstrate that glioblastoma cells relocate astrocytic end-feet along with endothelial cells [23]. AQP4 is originally arranged as orthogonal arrays of particles [24]. During glioblastoma progression, the array arrangement of this channel protein is disturbed. Instead, distribution of the AQP shifts from glial membranes in contact with the mesenchymal space to cover the entire surface of tumor cells [24]. Such a redistribution is associated with loss of agrin immune-reactivity from brain capillary basal laminae in human glioblastomas. Recently, Tome-Garcia et al. reported that repressing AQP4 expression by knocking-down transcriptional factor transcriptional enhancer factor 1/4 concurrently defeats migration of human glioblastoma cells [25]. In addition, Monti et al. showed that levels of AQP4 decreased in mice lacking BDKRB2 [26]. Bradykinin, an agonist of BDKRB1/2, can induce calcium influx from the external environment into the cytoplasm and triggers activation of the mitogen-activated protein kinase (MAPK) pathway, including mitogen-activated protein kinase kinases (MEKs) and extracellular signal-regulated kinases (ERKs) [27]. In this study, we investigated the roles of the bradykinin-BDKRB1/B2 axis in regulating glioblastoma cell activities and the possible mechanisms from the viewpoint of calcium influx-induced signal-transducing events in glioblastomas.

## 2. Results 

### 2.1. Expressions of AQP4 and BDKRB1/2 mRNAs in Human Glioblastomas

**Figure 1:**
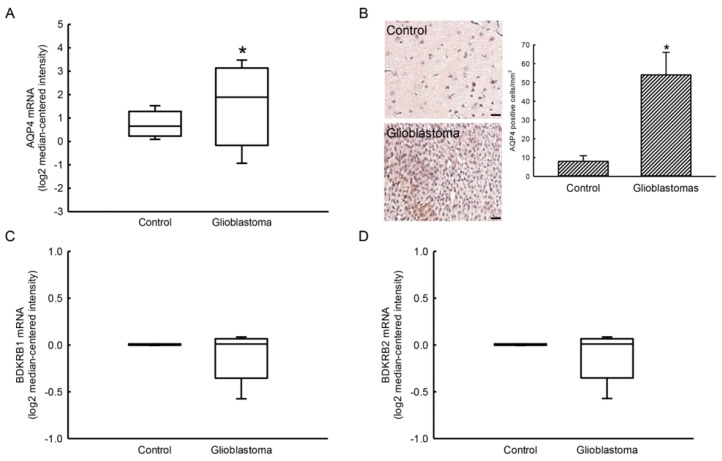
Expression of aquaporin-4 (AQP4) and bradykinin receptor (BDKR) B1/2 mRNAs or proteins in human glioblastomas and normal brain tissues. Expression of AQP4 mRNA in human normal brains (Control, *n* = 37) and glioblastomas (Glioblastoma, *n* = 542) was mined in The Cancer Genome Atlas (TCGA) database (**A**). An immunohistochemical analysis of AQP4 in human meningioma (Control) and glioblastoma (Glioblastoma) tissues was carried out (**B**). Representative images are shown. The signals were quantified and statistically analyzed (**C**). Each value represents the mean ± standard deviation (SD) for n = 3. Expression of BDKRB1/2 mRNAs from controls (*n* = 37) and glioblastomas (*n* = 582) were searched using TCGA cohort (**D**). An asterisk (*) indicates that a value significantly (*p* < 0.05) differed from the respective control. Scale bar, 50 μm.

Expressions of AQP4 and BDKRB1/2 mRNAs in human glioblastoma were mined in The Cancer Genome Atlas (TCGA) (Figure 1). Compared to human normal brain tissues, expression of AQP4 mRNA in human glioblastomas was induced 2.9-fold (Figure 1A). Our results by immunohistochemistry further reveal that levels of the AQP4 protein in human glioblastomas were elevated compared to human meningioma tissues (Figure 1B). The immunodetected signals were quantified and statistically analyzed (Figure 1C). In human glioblastomas, levels of AQP4 had increased by 6.8-fold. In contrast, expression of BDKRB1/2 mRNA was not altered compared to human normal brain tissues (Figure 1D).

### 2.2. Bradykinin Specifically Increased Levels of BDKRB1 and Stimulated Ca^2+^ Influx without Affecting Cell Survival in Human Malignant Glioblastoma Cells

**Figure 2:**
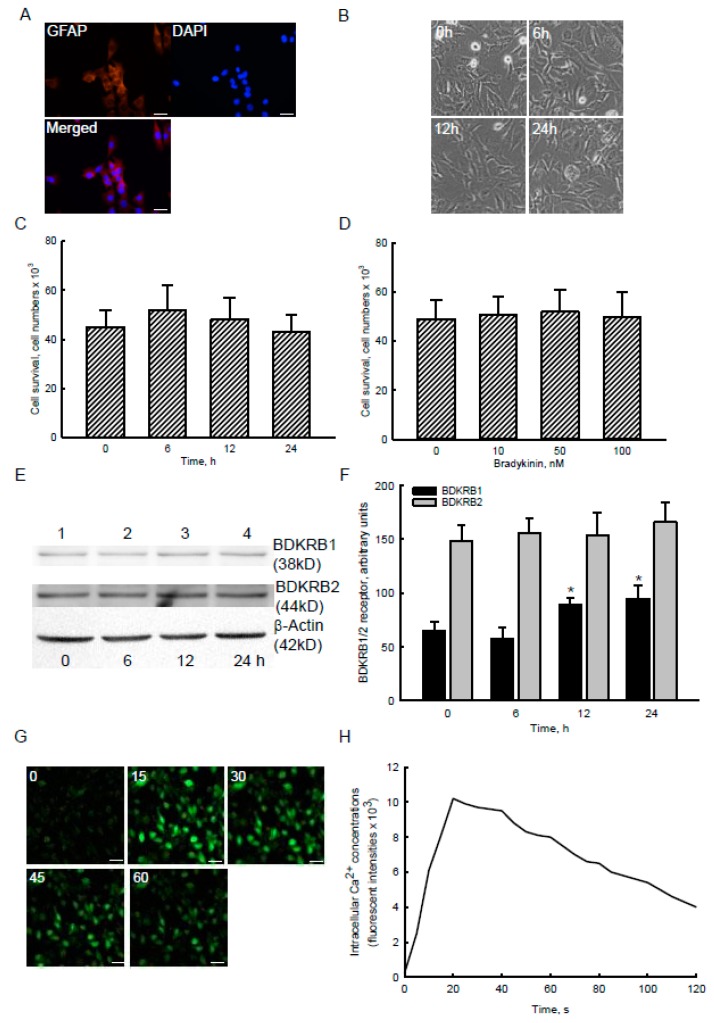
Effects of bradykinin on viability, levels, and functions of bradykinin receptor (BDKR) B1/2 in human malignant glioblastoma cells. Human U87 MG glioblastoma cells were stained with a fluorescent 4’,6-diamidino-2-phenylindole (DAPI) dye and reacted with a monoclonal antibody against glial fibrillary acidic protein (GFAP), a biomarker of astrocytes (**A**). Fluorescent signals were observed and analyzed using confocal microscopy. U87 MG cells were treated with 100 nM bradykinin for 6, 12, and 24 h or with 10, 50, and 100 nM bradykinin for 24 h. Cell morphologies were observed and photographed using a light microscope (**B**). Cell survival was analyzed using a trypan blue exclusion method (**C**,**D**). Levels of BDKRB1 and BDKRB2 were immunodetected (**E**, top two panels). β-Actin was analyzed as an internal control (bottom panel). These protein bands were quantified and statistically analyzed (**F**). After exposure to bradykinin and Fluo3, dynamic changes in levels of intracellular calcium (Ca^2+^) were immediately observed and recorded by confocal microscopy (**G**). Marked enhancement of fluorescent signals showed the increased intensities of intracellular Ca^2+^ following bradykinin treatment (**H**). Each value represents the mean ± standard deviation (SD) for n = 9. Representative immunoblots and confocal images are shown. An asterisk (*) indicates that a value significantly (*p* < 0.05) differed from the respective control. Scale bar, 20 μm.

Immunocytochemical images show the expression of glial fibrillary acidic protein (GFAP), a biomarker of astrocytes, in human U87 MG glioblastoma cells (Figure 2A, left panel). Nuclei were stained with DAPI (middle panel). Merged signals show that GFAP was detected in the cytoplasm of human U87 MG cells (bottom panel). After exposure to 100 nM bradykinin for 6, 12, and 24 h, morphologies of human U87 MG glioblastoma cells did not change (Figure 2B). An assay of cell survival displayed that treatment of human U87 MG cells with 100 nM bradykinin for 6, 12, and 24 h or with 10, 50, and 100 nM bradykinin for 24 h did not cause cell death (Figure 2C,D). Levels of BDKRB1 and BDKRB2 were detected in human U87 MG glioblastoma cells (Figure 2E, top two panels, lane 1). Compared to untreated glioblastoma cells, exposure to 100 nM bradykinin for 12 and 24 h increased levels of BDKRB1 (lanes 3 and 4). However, bradykinin did not influence levels of BDKRB2 in human U87 MG cells (lanes 2~4). Amounts of β-actin were examined as an internal control (bottom panel). These immunoreacted protein bands were quantified and statistically analyzed (Figure 2F). Treatment of human U87 MG glioblastoma cells with 100 nM bradykinin for 12 and 24 h led to significant 37% and 45% augmentations in levels of the BDKRB1 protein.

Analysis by confocal microscopy showed that levels of intracellular Ca^2+^ in human U87 MG glioblastoma cells were massively augmented following exposure to 100 nM bradykinin for 15 s (Figure 2G). Compared to the high peak signals at 15 s, the bradykinin-induced augmentation of Ca^2+^ influx in human U87 MG cells time-dependently decreased after exposure for 30, 45, and 60 s (Figure 2G). The fluorescent signals were quantified and statistically analyzed (Figure 2H). Exposure to 100 nM bradykinin for 15 s led to a 44-fold increase in levels of intracellular Ca^2+^ in human malignant glioblastoma cells. In contrast, signals of Ca^2+^ influx reached a high peak of a 44-fold increase following exposure to bradykinin for 20 s. Nonetheless, after treatment with bradykinin for 30, 45, 60, 90, and 120 s, levels of intracellular calcium had increased by 42-, 38-, 35-, 25-, and 17-fold, respectively (Figure 2H).

### 2.3. Bradykinin Successively Activated MEK1 and ERK1/2 in Human Malignant Glioblastoma Cells

**Figure 3:**
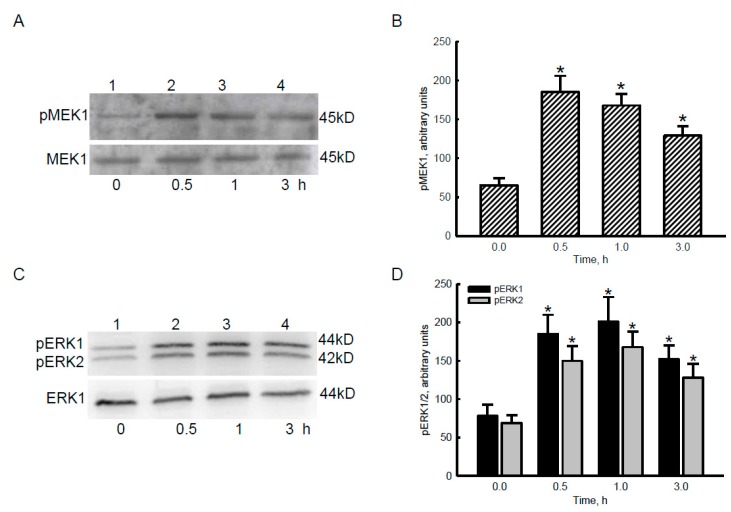
Effects of bradykinin on phosphorylation of mitogen-activated protein kinase kinase (MEK)1 and extracellular signal-regulated kinase (ERK1)/2 in human malignant glioblastoma cells. Human U87 MG glioblastoma cells were treated with 100 nM bradykinin for 0.5, 1, and 3 h. Levels of phosphorylated (p)-MEK1and p-ERK1/2 were immunodetected (**A**,**C**, top panels). Amounts of MEK1 and ERK1 were analyzed as the internal controls (bottom panels). These protein bands were quantified and statistically analyzed (**B**,**D**). Each value represents the mean ±standard deviation (SD) for n = 9. An asterisk (*) indicates that a value significantly (*p* < 0.05) differed from the respective control. Representative immunoblots are shown.

To determine the consequent effects of Ca^2+^ influx stimulated by the bradykinin-BDKR1/2 axis, phosphorylation of MEK1 and ERK1/2 was immunologically examined in human malignant glioblastoma cells (Figure 3). Compared to the control group, exposure of human U87 MG cells to 100 nM bradykinin for 30 min caused an obvious increase in levels of p-MEK1 (Figure 3A, top panel, lane 2). Phosphorylation of MEK1 was also enhanced following bradykinin treatment for 1 and 3 h (lanes 3 and 4). MEK1 was immunodetected as an internal standard (bottom panel). These protein bands were quantified and statistically analyzed (Figure 3B). Exposure of human U87 MG cells to bradykinin for 0.5, 1, and 3 h led to 2.8-, 2.6-, and 2-fold augmentation in levels of p-MEK1, respectively.

In parallel with MEK1 activation, treatment of human U87 MG cells with 100 nM bradykinin for 30 min caused obvious increases in the phosphorylation of ERK1 and ERK2 (Figure 3C). After exposure to bradykinin for 1 and 3 h, levels of p-ERK1 and p-ERK2 were also enhanced (lanes 3 and 4). ERK1 was analyzed as an internal standard (bottom panel). These protein bands were quantified and statistically examined (Figure 3D). Treatment with bradykinin for 0.5, 1, and 3 h respectively led to 137%, 158%, and 95% increases in levels of p-ERK1 in human malignant glioblastoma cells. By comparison, exposure to bradykinin for 0.5, 1, and 3 h enhanced phosphorylation of ERK2 in human U87 MG cells by 117%, 143%, and 86%, respectively (Figure 3D).

### 2.4. Bradykinin Accordingly Triggered Translocation and Transactivation Activity of NF-κB in Human Malignant Glioblastoma Cells

**Figure 4:**
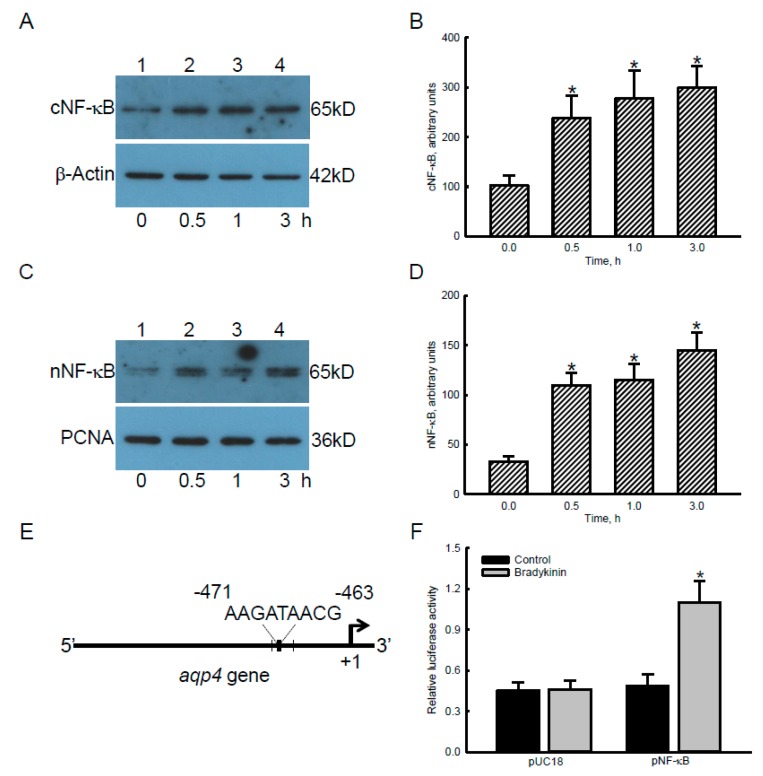
Effects of bradykinin on levels, translocation, and transactivation activity of nuclear factor-kappaB (NF-κB) in human malignant glioblastoma cells. Human U87 MG glioblastoma cells were treated with 100 nM bradykinin for 0.5, 1, and 3 h. Levels of cytosolic (c) and nuclear (n) NF-κB were immunodetected (**A**,**C**, top panels). Amounts of β-actin and proliferating cell nuclear antigen (PCNA) were analyzed as internal controls for the cytosolic and nuclear proteins, respectively (bottom panels). These protein bands were quantified and statistically analyzed (**B**,**D**). A schematic diagram indicates the NF-κB-specific DNA binding element (−463 to −471) in the 5’-promoter region of the *aqp4* gene (**E**). The NF-κB luciferase reporter plasmids (pNF-κB) and pUC18 control plasmids (pUC18) were transfected into human U87 MG cells. Transactivation activity of NF-κB was assayed with a reporter gene assay (**F**). Each value represents the mean ± standard deviation (SD), n = 9. An asterisk (*) indicates that a value significantly (*p* < 0.05) differed from the respective control. Representative immunoblots are shown.

Bradykinin-triggered signal-transducing events were further investigated in human glioblastoma cells (Figure 4). Compared to the control group, exposure of human U87 MG glioblastoma cells to 100 nM bradykinin for 0.5, 1, and 3 h led to noteworthy increases in levels of cytosolic and nuclear NF-κB (Figure 4A,C, top panels, lanes 2~4). Levels of cytosolic β-actin and nuclear PCNA were analyzed as internal controls (bottom panels). These protein bands were quantified and statistically analyzed (Figure 4B,D). Treatment of human U87 MG cells with bradykinin for 0.5, 1, and 3 h caused 2.3-, 2.7-, and 2.9-fold enhancements in cytosolic NF-κB (Figure 4B). Levels of nuclear NF-κB in human glioblastoma cells were augmented by 3.3-, 3.5-, and 4.4-fold following exposure to bradykinin for 0.5, 1, and 3 h, respectively (Figure 4D).

Our bioinformatics results revealed that at least five NF-κB-specific DNA-binding elements exist in the 5′-promoter region of the *aqp4* gene (Figure 4E). After transfection of blank pUC18 plasmids into human U87 MG cells, treatment with bradykinin did not affect production of luminance signals (Figure 4F). Nonetheless, exposure of human glioblastoma U87 MG cells transfected with NF-κB luciferase reporter plasmids to bradykinin caused a significant 2.4-fold increase in luciferase activity (Figure 4F).

### 2.5. Bradykinin Induced AQP4 mRNA and Protein Expression via Activation of BDKRB1

**Figure 5:**
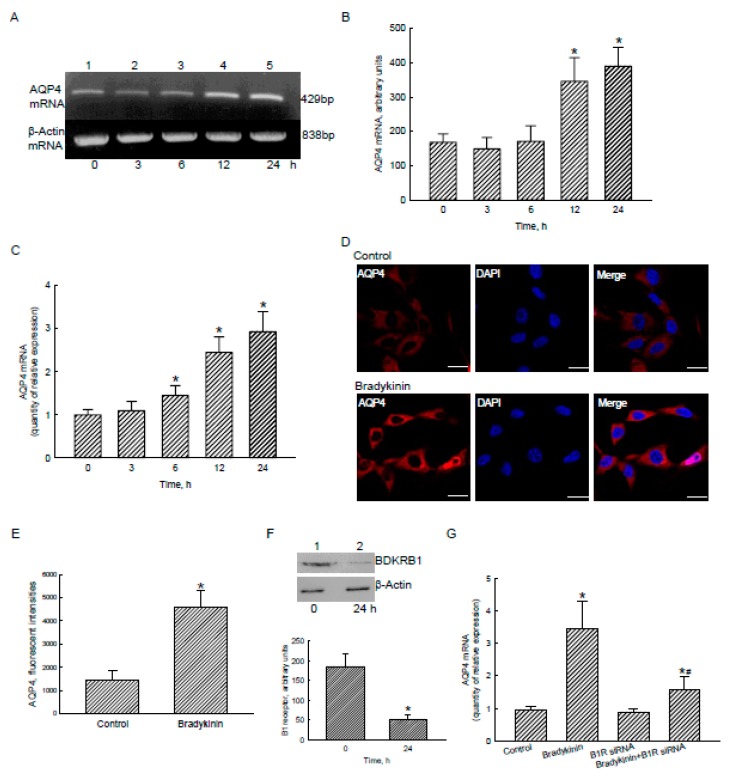
Effects of bradykinin on aquaporin-4 (AQP4) mRNA and protein expressions in human malignant glioblastoma cells. Human U87 MG glioblastoma cells were treated with 100 nM bradykinin for 3, 6, 12, and 24 h. Levels of AQP4 mRNA were analyzed using an RT-PCR (**A**, top panel). Amounts of β-actin mRNA were examined as an internal control (bottom panel). These DNA bands were quantified and statistically analyzed (**B**). Expression of AQP4 mRNA was further quantified using a real-time polymerase chain reaction (PCR) analysis (**C**). Human U87 MG cells were exposed to bradykinin for 24 h. Levels and distribution of AQP4 were immunodetected (**D**, left panel). The nucleus was stained with 4’,6-diamidino-2-phenylindole (DAPI) (middle panel). The merged signals are shown in the right panel (**D**) and were quantified and statistically analyzed (**E**). Expression of the bradykinin receptor (BDKR) B1 was knocked-down using RNA interference. Control cells received scrambled siRNA. Levels of BDKRB1 were immunodetected (**F**, top panel). β-Actin was immunodetected as an internal control. These protein bands were quantified and statistically analyzed (bottom panel). Human U87 MG cells were pretreated with BDKRB1 siRNA and then exposed to bradykinin. Expression of AQP4 mRNA was quantified with a real-time PCR (**G**). Each value represents the mean ± standard deviation (SD), n = 9. Symbols * and ^#^ indicate that the values significantly (*p* < 0.05) differed from the control and BDKRB1 siRNA-treated groups, respectively. Representative DNA agarose gels, confocal images, and immunoblots are shown. Scale bar, 5 μm.

To confirm the roles of the bradykinin-BDKR1/2 axis-induced NF-κB translocation and transactivation in regulating *aqp4* gene expression, RNA and protein analyses were further carried out (Figure 5). In untreated human U87 MG glioblastoma cells, AQP4 mRNA was detected (Figure 5A, top panel, lane 1). Treatment of human U87 MG cells with 100 nM bradykinin for 3 and 6 h did not change AQP4 mRNA expression (lanes 2 and 3). However, exposure to bradykinin for 12 and 24 h perceptibly induced the expression of AQP4 mRNA (lanes 4 and 5). β-Actin mRNA was analyzed as an internal control (bottom panel). These DNA bands were quantified and statistically analyzed (Figure 5B). Exposure to bradykinin for 12 and 24 h respectively caused significant 2.1- and 2.3-fold induction of AQP4 mRNA expression in human malignant glioblastoma cells. To prove the effects of bradykinin on AQP4 mRNA expression, a real-time PCR was conducted (Figure 5C). Treatment of human U87 MG cells with 100 nM bradykinin for 6, 12, and 24 h, respectively, led to 45%, 145%, and 193% increases in levels of AQP4 mRNA.

Analysis by confocal microscopy showed that basic levels of the AQP4 protein were detected (Figure 5D, left-top panel). In contrast, exposure to 100 nM bradykinin for 24 h elevated amounts of AQP4 in human U87 MG glioblastoma cells (left-bottom panel). Nuclei of glioblastoma cells were stained with DAPI dye (middle panels). The merged images show that bradykinin-induced AQP4 was localized in the cytoplasm and plasma membranes (right panels). Fluorescent signals were quantified and statistically analyzed (Figure 5E). Treatment with bradykinin caused a 3.2-fold augmentation in the AQP4 protein in human U87 MG cells.

An RNAi analysis was conducted to evaluate the role of the BDKRB1 in bradykinin-induced AQP4 mRNA expression. Application of BDKRB1 siRNA to human U87 MG cells led to a substantial reduction in levels of the AQP4 protein (Figure 5F, top panel). β-Actin was immunodetected as an internal control. These protein bands were quantified and statistically analyzed (bottom panel). Knocking-down BDKRB1 using RNAi decreased translation of this receptor by 72%. Exposure to bradykinin induced AQP4 mRNA expression in human malignant glioblastoma cells by 3.6 fold (Figure 5G). Application of BDKR1 siRNA to human U87 MG cells did not influence AQP4 mRNA expression. In contrast, knocking-down BDKRB1 caused a significant 76% inhibition in bradykinin-induced AQP4 mRNA expression (Figure 5G).

### 2.6. Bradykinin Stimulated Migration and Invasion of Human Malignant Glioblastoma Cells via Activation of BDKRB1

**Figure 6:**
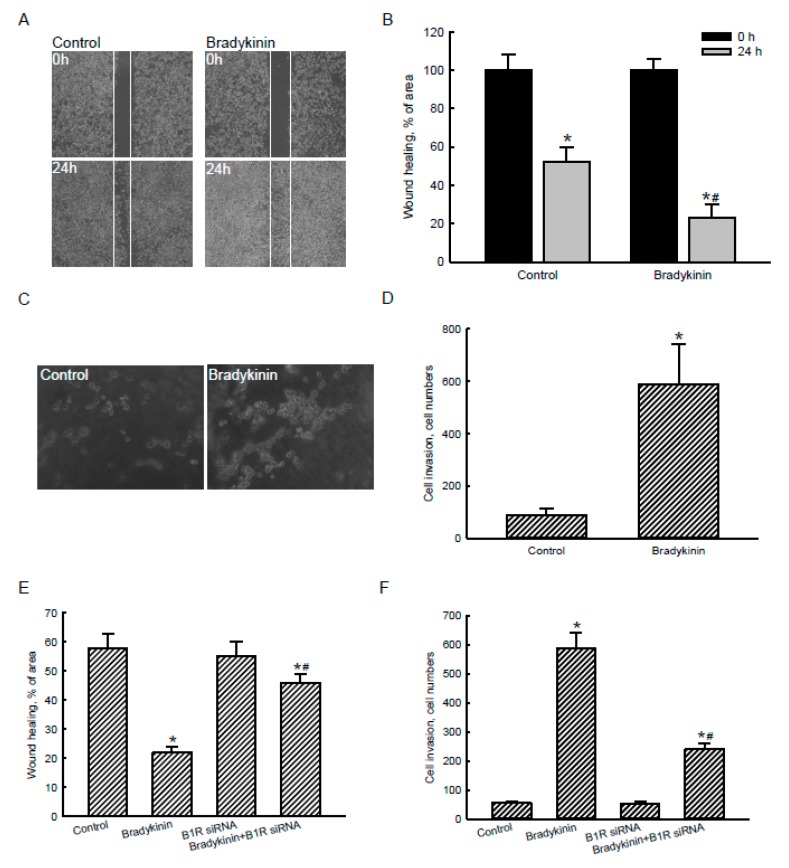
Effects of bradykinin on the migration and invasion of human malignant glioblastoma cells. Human U87 MG glioblastoma cells were treated with 100 nM bradykinin for 24 h. A wound-healing assay was carried out to determine migration of U87 MG cells (**A**). Cell migration was quantified by analyzing the blank area (**B**). In parallel, invasion by U87 MG cells was further analyzed using a Matrigel-based invasion assay (**C**). Invading cells were counted and statistically analyzed (**D**). Human U87 MG cells were pretreated with bradykinin receptor B1 (BDKRB1) siRNA and then exposed to bradykinin. Wound-healing (**E**) and Matrigel-invasion (**F**) assays were performed, and results were statistically analyzed. Each value represents the mean ± standard deviation (SD), *n* = 9. Symbols * and ^#^ indicate that the values significantly (*p* < 0.05) differed from the control and BDKRB1 siRNA-treated groups, respectively. Representative morphological images are shown.

Effects of bradykinin-induced AQP4 expression on the migration and invasion of human malignant glioblastoma cells were evaluated using wound-healing and Matrigel-based invasion assays (Figure 6). The results of a wound-healing assay revealed that after culturing human U87 MG cells for 24 h, tumor cells had migrated and expanded into a cell-scraped area (Figure 6A, left panels). In contrast, treatment of human malignant U87 MG glioblastoma cells with 100 nM bradykinin for 24 h obviously stimulated cell migration and occupation of the blank space (right panels). These occupied cells were quantified and statistically analyzed (Figure 6B). Compared to the control group, exposure of human U87 MG glioblastoma cells to bradykinin caused a 2.3-fold enhancement in wound-healing activity. Results of a Matrigel-based invasion assay showed that after culturing for 24 h, human U87 MG glioblastoma cells had invaded the bottom layer of the transwell (Figure 6C, left panel). In contrast, treatment of human glioblastoma cells with bradykinin obviously increased cell invasion (right panel). These invading cells were quantified and statistically analyzed (Figure 6D). The invasive capacity of human malignant U87 MG glioblastoma cells was elevated 6-fold following administration of bradykinin (Figure 6D).

Exposure of human U87 MG glioblastoma cells to 100 nM bradykinin for 24 h triggered 2.6-fold wound-healing activity (Figure 6E). Application of BDKRB1 siRNA to human glioblastoma cells did not affect the wound-healing activity. In comparison, knocking-down translation of the BDKRB1 gene caused a 60% reduction in the bradykinin-induced augmentation of wound-healing activity (Figure 6E). Furthermore, invasion by human malignant U87 MG glioblastoma cells increased 11-fold due to bradykinin (Figure 6F). Application of BDKRB1 siRNA did not change cell invasion. Nevertheless, suppressing translation of the BDKRB1 gene using RNAi concurrently decreased the bradykinin-triggered invasion by human malignant U87 MG glioblastoma cells (Figure 6F).

### 2.7. The Bradykinin-Induced Ca^2+^ Influx, AQP4 mRNA Expression, Wound Healing, and Cell Migration and Invasion Were Further Confirmed in Murine GL261 Glioblastoma Cells

The effects of bradykinin on regulating activities of malignant glioblastoma cells were further confirmed in murine GL-261 glioblastoma cells (Table 1). Treatment of murine GL-261 glioblastoma cells with 100 nM bradykinin did not affect cell survival. In contrast, exposure to bradykinin led to a 20-fold increase in levels of intracellular calcium in murine glioblastoma cells (Table 1). Successively, expression of AQP4 mRNA in murine GL-261 glioblastoma cells was induced by 3.2-fold following exposure to bradykinin. Consequently, treatment of murine GL-261 glioblastoma cells with bradykinin caused a 2.5-fold augmentation in wound-healing activity. Moreover, exposure to bradykinin led to a significant 12-fold elevation in invasion of murine malignant GL-261 glioblastoma cells (Table 1).

## 3. Discussion

This study showed that the bradykinin-BDKRB1/2 axis contributes to migration and invasion of malignant glioblastoma cells through regulation of *aqp4* gene expression. The signal-transducing events were involved in activation of the Ca^2+^ influx-MAPK-NF-κB pathway. Previous studies showed that bradykinin can be massively produced in glioblastomas through activation of the KKS system [13,14]. Functionally, accumulation of bradykinin is highly associated with consistent progression of brain tumors [15,16,17]. Herein, administration of malignant glioblastoma cells with bradykinin significantly stimulated Ca^2+^ influx and subsequent activation of the MAPK pathway. In parallel, bradykinin induced AQP4 mRNA and protein expressions. Fascinatingly, knocking-down AQP4 expression concurrently led to the suppression of migration and invasion of glioblastoma cells. Ding et al. reported an association of AQP4 downregulation with death of glioblastoma cells [22]. Speedy migration and invasion of glioblastoma cells into surrounding healthy brain tissues are two typical characteristics leading to poor outcomes for GBM patients [7]. Nico et al. reported an enormous increase in AQP4 in glioblastomas [21]. Our present study showed upregulation of AQP4 mRNA and protein expressions in human glioblastomas. Additionally, the enhancement of AQP4 is highly associated with tumor-related brain edema. Recently, AQP4 was functionally shown to regulate migration of glioblastoma cells [25]. Hence, AQP4 may participate in the development of brain tumors. In GBM, inflammation and hypoxic conditions can stimulate the production and accumulation of bradykinin, the most protuberant kininogen of the KKS system [13]. Montana et al. demonstrated that bradykinin induces the chemotactic invasion of primary brain tumors [14]. This study further demonstrated that the bradykinin-BDKRB1/2 axis takes part in the rapid migration and invasion of glioblastoma cells due to induction of *aqp4* gene expression.

Bradykinin can trigger BDKRB1/2 and then stimulate Ca^2+^ influx in glioblastoma cells. In this study, we showed that GFAP, a biomarker of astrocytes, was expressed in human U87 MG glioblastoma cells. Exposure of U87 MG cells to bradykinin did not affect cell survival or proliferation. Bradykinin functions as a specific ligand of the BDKRB1/2 [18,19]. In human glioblastomas and normal brain tissues, basal levels of BDKRB1/2 mRNA were detected. Also, we demonstrated the existence of BDKRB1/2 proteins in human malignant glioblastoma cells. After exposure to bradykinin, levels of BDKRB1 were significantly augmented. Interestingly, exposure to bradykinin stimulated the influx of Ca^2+^ into human glioblastoma cells from the extracellular medium. In the tumorigenesis of brain tumors, bradykinin functions as an inflammatory factor to stimulate cell proliferation and interactions between glioblastoma cells and mesenchymal stem cells [14,15]. Bradykinin can increase the permeability of the blood-tumor barrier in GBM [19]. A clinical trial used RMP-7, an agonist of bradykinin, to increase permeability of the blood-tumor barrier for entrance of carboplatin, a chemotherapeutic agent, into tumor sites for GBM therapy [18]. Therefore, bradykinin is involved in tumorigenesis of GBM through stimulating Ca^2+^ influx due to binding and activation of BDKRB1/2.

Bradykinin subsequently stimulated phosphorylation of MEK1 and ERK1/2 in glioblastoma cells. In parallel with Ca^2+^ influx, exposure of human malignant glioblastoma cells to bradykinin time-dependently induced phosphorylation of MEK1, a critical member of MAPK cascade proteins. MEK1 acts as a second central signaling module to transduce extracellular information into the cytoplasm and nuclei [27]. In the formation and maturation of neuronal networks, MEK1-involved MAPK signal-transducing events are evoked by Ca^2+^ influx and then contribute to nuclear plasticity and geometry [28]. Hence, one of the possible causes explaining bradykinin-induced MEK1 phosphorylation is BDKRB1/2-mediated Ca^2+^ influx. ERK1/2 are downstream targets of MEK1 [27]. This study showed that bradykinin sequentially increased the phosphorylation of MEK1 and ERK1/2 in human glioblastoma cells. The MEK-ERK signaling pathway was involved in inflammation-associated processes in GBM, including tumor-associated macrophage signatures [29]. Thus, bradykinin-induced activation of MEK1-ERK1/2 directly participates in tumorigenesis of glioblastomas by transducing information from the site of Ca^2+^ signal generation in plasma membranes to nuclei.

The NF-κB transcriptional factor takes part in bradykinin-induced signal-transducing events in glioblastomas. Treatment of human malignant glioblastoma cells with bradykinin increased levels of cytosolic NF-κB. Simultaneously, levels of nuclear NF-κB in human U87 MG cells were augmented following exposure to bradykinin. ERK1/2 is an upstream kinase that can specifically activate NF-κB and stimulates its translocation to nuclei [30]. Thus, bradykinin can trigger translocation of NF-κB from the cytoplasm to nuclei via activating the MEK1-ERK1/2 cascade mechanism. NF-κB extensively participates in numerous cellular processes such as differentiation, proliferation, survival, and motility through regulating a wide array of gene expressions [30]. In response to a variety of stimuli in the growth of glioblastomas, NF-κB can be emergently activated for migration, invasion, chemoresistance, resistance to radiotherapy, GBM stemness, and mesenchymal identity [31]. Terzuoli et al. demonstrated involvement of the bradykinin-NF-κB axis in angiogenesis and inflammatory responses in human endothelial cells [32]. The present study further showed the roles of the BDKRB1/2-MAPK-NF-κB axis in the tumorigenesis of glioblastomas.

The bradykinin-BDKRB1 axis is involved in regulating the *aqp4* gene in glioblastoma cells via activating the MAPK-NF-κB mechanism. AQP4, an aquaporin water channel protein, controls homeostasis of the microenvironment in plasma membranes by guiding a Ca^2+^-dependent gating of the water channel [20]. In motor neurons, alterations in AQP4 expression and polarization lead to a widespread pathological condition [33]. In human glioblastomas, levels of AQP4 mRNA and protein are upregulated. In addition, our present results proved that bradykinin can induce *aqp4* gene expression in human and murine glioblastoma cells. Moreover, our confocal microscopic images showed that the distribution of elevated AQP4 was mainly located in the cytoplasm and plasma membranes of malignant glioblastoma cells. Nico et al. described a positive role of AQP4 in regulating edema production in glioblastomas [21]. In the present study, we showed the potential action of AQP4 on producing edema in brain tumors due to accumulation of bradykinin. As to the mechanisms, the bioinformatics results revealed the existence of NF-κB-specific binding elements in the 5’-promoter region of the *aqp4* gene. Furthermore, results of a reporter gene assay provided direct evidence proving involvement of NF-κB in bradykinin-induced *aqp4* gene expression in human glioblastoma cells. Thus, bradykinin can induce *aqp4* gene expression via a MEK4-ERK1/2-NF-κB-dependent mechanism. More fascinatingly, by knocking-down BDKRB1, bradykinin-induced *aqp4* gene expression was concurrently inhibited. Following cortical stab injuries, the MAPK-signaling pathway is involved in inducing AQP1 expression in astrocytes [34]. In comparison, this study provides a whole mechanism regarding bradykinin-induced *aqp4* gene expression in glioblastomas via activation of the BDKRB1-MEK4-ERK1/2-NF-κB axis.

The bradykinin-BDKRB1 axis participates in migration and invasion of glioblastoma cells. In this study, we showed that exposure to bradykinin caused significant enhancements in migration and invasion of glioblastoma cells. Previous studies showed the effects of bradykinin on stimulating cancer cells. For example, bradykinin can trigger proliferation, migration, and invasion of gastric cancer cells and cervical cancer cells via activation of the ERK and signal transducer and activator of transcription (STAT)-3 signaling pathways [35,36]. In hepatocellular carcinoma cells, bradykinin can trigger tumor metastasis [37]. In this study, we showed the effects of bradykinin on elevation of BDKRB1 in human glioblastoma cells. Remarkably, knocking-down BDKRB1 concurrently attenuated bradykinin-induced migration and invasion of brain tumor cells. Selective antagonists for BDKRB1 possess antiproliferative, anti-inflammatory, antiangiogenic and anti-migratory properties [38]. Wang et al. reported that suppressing BDKRB2, but not BDRKB1, decreased interleukin-6 production and thereby reduced the invasion and migration of colorectal cancer cells [35]. Thus, this study demonstrated the contribution of the bradykinin-BDKRB1 axis to migration and invasion of human and murine glioblastoma cells. BDKRB1 antagonists may interfere less with housekeeping functions and hence could be attractive compounds to treat selected types of cancers [38]. The present study showed overexpression of AQP4 in human glioblastomas and involvement of the bradykinin-BDKRB1 axis in regulating expression of this channel gene. Upregulation of AQP4 can disturb the arrangement of orthogonal arrays of particles on the entire surface of tumor cells, resulting in changes in cytoskeletal and morphological structures [24]. Interestingly, repressing AQP4 expression concurrently abrogated migration of human glioblastoma cells [25]. Rapid migration and invasion are critical factors driving the malignance of glioblastoma cells [7]. As a result, the bradykinin-BDKRB1 axis-induced AQP4 plays an important role in the migration and invasion of glioblastoma cells.

## 4. Materials and Methods

### 4.1. Reagents

Dulbecco’s modified Eagle’s medium (DMEM), fetal bovine serum (FBS), and trypsin- ethylenediaminetetraacetic acid (EDTA) were purchased from Gibco-BRL (Grand Island, NY, USA). Bradykinin, 3-(4,5-dimethylthiazol-2-yl)-2,5-diphenyltetrazolium bromide (MTT), 4′,6-diamidino-2-phenylindole, and dimethyl sulfoxide (DMSO) were obtained from Sigma (St. Louis, MO, USA). A bicinchonic acid protein assay kit and cytoplasmic and nuclear extraction kits were purchased from Pierce (Rockford, IL, USA). Rabbit BDKRB1 (Cat no. ADI-905-787-100; 100 μg/100 μL, 1:500 dilution) and BDKRB2 (Cat no. NBP1-46328; 100 μg/100 μL, 1:500 dilution) polyclonal antibody (pAb) were acquired from Enzo Life Sciences (Farmingdale, NY, USA) and Novus Biologicals (Littleton, CO, USA), respectively. Phosphorylated (p)-MEK1 (Cat no. 9121; 100 μg/100 μL, 1:1000 dilution), p-ERK1/2 (Cat no. 4370; 100 μg/100 μL, 1:1000 dilution), and MEK1 (Cat no. 9124; 1:1000 dilution) pAbs were purchased from Cell Signaling (Danvers, MA, USA). Rabbit AQP4 (Cat no. sc-20812; 200 μg/mL, 1:200 dilution), ERK1 (Cat no. sc154; 200 μg/ml, 1:200 dilution), NF-κB (Cat no. Sc372, 200 μg/mL, 1:200 dilution), and proliferating cell nuclear antigen (PCNA) (Cat no. sc7907; 1:200 dilution) pAbs were bought from Santa Cruz Biotechnology (Santa Cruz, CA, USA). Mouse β-Actin (Cat no. 1978; 2mg/mL, 1:1000 dilution) monoclonal antibody (mAb) was bought from Sigma. Fluo-3/AM was obtained from Invitrogen (Carlsbad, CA, USA). Biotin-SP-conjugated AffiniPure goat anti-rabbit immunoglobulin G and a third antibody with Cy3-conjugated streptavidin were acquired from Jackson ImmunoResearch (West Grove, PA, USA). NF-κB luciferase reporter plasmids and a FuGene transfection reagent were purchased from Stratagene (La Jolla, CA, USA) and Promega (Madison, WI, USA), respectively. B1 bradykinin receptor small interfering (si)RNA was purchased from Santa Cruz Biotechnology. Matrigel was from BD Biosciences (San Jose, CA, USA).

### 4.2. Data Downloading and Preprocessing

Expressions of AQP4, BDKRB1, and BDKR2 messenger (m)RNAs in human normal brain tissues (Figure 1, Control group) and glioblastomas (Figure 1, Glioblastoma group) were analyzed by mining The Cancer Genome Atlas (TCGA) database (https://www.cancer.gov/about-nci/organization/ccg/research/structural-genomics/tcga, with data downloaded in December 2019). mRNA expression data were selected for downloading in the Fragments Per Kilobase Million (FPKM) format.

### 4.3. Immunohistochemical (IHC) Analysis of AQP4

An immunohistochemical (IHC) assay was carried out following the method as described previously [39]. Our study was approved by the joint-institutional review board of Taipei Medical University (N201903060). Specimens of human glioblastoma and normal brain tissues were identified and acquired from Department of Pathology, Shuang Ho Hospital, Taipei Medical University, Taipei, Taiwan. These brain specimens were incubated with 0.2% Triton X-100. A mouse mAb against human AQP4 was used for IHC detection of AQP4.

### 4.4. Culture of Malignant Glioblastoma Cells and Drug Treatments

The human malignant U87 MG glioblastoma cell line was purchased from American Type Culture Collection (Manassas, VA, USA). Murine GL261 glioblastoma cells were a kind gift from Dr. Rong-Tsun Wu (Institute of Biopharmaceutical Sciences, National Yang-Ming University, Taipei, Taiwan). These two types of glioblastoma cells were grown in DMEM accompanied by 10% FBS, 2 mM L-glutamine, 100 IU/mL penicillin, 100 mg/mL streptomycin, 1 mM sodium pyruvate, and 1 mM nonessential amino acids at 37 °C in a humidified atmosphere of 5% CO_2_. These human and murine glioblastoma cells were grown to confluence before drug treatment as described previously [40]. Glioblastoma cells were treated with 10, 50, and 100 nM bradykinin for 6, 12, and 24 h.

### 4.5. Cell Morphology and Cell Survival

The toxicity of bradykinin to human and murine malignant glioblastoma cells was determined by analyzing the cell morphology and cell survival. Glioblastoma cells (10^4^ cells/well) were seeded in tissue culture plates overnight and then treated with 10, 50, and 100 nM bradykinin for 6, 12, and 24 h. Morphologies of glioblastoma cells were observed and photographed using a reverse-phase microscope (Nikon, Tokyo, Japan) as described previously [41]. Cell survival was assayed using a trypan blue exclusion method [42]. After exposure to bradykinin, glioblastoma cells were trypsinized with 0.1% trypsin-EDTA. After centrifugation, cells were suspended in phosphate-buffered saline (PBS) buffer (0.14 M NaCl, 2.6 mM KCl, 8 mM Na_2_HPO_4_, and 1.5 mM KH_2_PO_4_), and stained with trypan blue dye. Fractions of living cells without a blue signal were visualized and counted with a light microscope (Nikon). 

### 4.6. Immunoblot Analyses

Levels of BDKRB1/2, phosphorylated and non-phosphorylated MEK1 and ERK1/2, and β-actin were immunodetected as described previously [43]. After drug administration, glioblastoma cells were washed with PBS, and cell lysates were prepared in ice-cold radioimmunoprecipitation assay (RIPA) buffer (25 mM Tris-HCl (pH 7.2), 0.1% sodium dodecylsulfate (SDS), 1% Triton X-100, 0.15 M NaCl, and 1 mM EDTA). A mixture of proteinase inhibitors, including 1 mM phenylmethylsulfonyl fluoride, 1 mM sodium orthovanadate, and 5 µg/mL leupeptin, was added to the ice-cold RIPA buffer to avoid protein degradation. Protein concentrations were quantified by a bicinchonic acid protein assay kit (Pierce). Proteins at 100 µg per well were subjected to SDS-polyacrylamide gel electrophoresis (PAGE), and transferred to nitrocellulose membranes. Membranes were blocked with 5% non-fat milk at 37 °C for 1 h. BDKRB1, BDKRB2, p-MEK1, p-ERK1/2, MEK1, and ERK1 were immunodetected using rabbit pAbs. Mouse β-Actin was detected a mAb. Intensities of the immunoreactive bands were determined using a digital imaging system (UVtec, Cambridge, UK) as described previously [44]. Immunoblotting analyses were carried out for at least 3 determinations (the whole bolt can be found at supplementary). Intensities of BDKRB1 and BDKRB2 protein bands were normalized and quantified using β-actin as the internal control. Additionally, intensities of pMEK1 and pERK1/2 protein bands were normalized and quantified using MEK1 and ERK1 as the internal controls, respectively.

### 4.7. Extraction and Immunodetection of Nuclear Proteins

Nuclear proteins were extracted and prepared following a previously described method [45]. Briefly, after bradykinin treatment, human malignant U87 MG cells were collected, and cell lysates were prepared by reacting cells with ice-cold cytoplasmic extractions (Pierce) on ice for 10 min. After centrifugation at 15,000× *g* for 5 min, the fraction of a nuclear pellet was reacted with ice-cold nuclear extraction reagent (Pierce) on ice for 40 min. Following centrifugation at 15,000× *g* for 10 min, the nuclear extract portion of a supernatant was collected. Protein concentrations were quantified with a bicinchonic acid protein assay kit (Pierce). Cytosolic and nuclear proteins (50 µg per well) were subjected to SDS-PAGE and then transferred to nitrocellulose membranes. After blocking, nuclear and cytosolic NF-κB levels were immunodetected using a rabbit pAb against mouse NF-κB. PCNA, a processivity factor for DNA polymerase, is a housekeep gene so it can be used as a loading control in immunoblotting analyses of nuclear proteins [45,46]. Cytosolic β-actin and nuclear PCNA were analyzed as the internal controls. Intensities of the immunoreactive bands were determined using a digital imaging system (UVtec). Immunoblotting analyses of cytosolic and nuclear NF-κB were carried out for at least three determinations. Intensities of cytosolic and nuclear NF-κB were normalized and quantified using β-actin and PCNA as the internal controls, respectively.

### 4.8. Measurement of Ca^2+^ Influx

Mobilization of Ca^2+^ from the extracellular medium into human U87 MG glioblastoma cells was analyzed as described previously [47]. U87 MG cells at a density of 7 × 10^4^ cells were grown on glass coverslips overnight, and then loaded with 4 μM Fluo-3/AM (Invitrogen), an indicator of Ca^2+^. Following administration of bradykinin, glioblastoma cells were immediately illuminated under a confocal laser scanning microscope (Olympus, Tokyo, Japan). Fluoview software (Olympus) was used to acquire and analyze images. Real-time images and fluorescent signals could be filmed and recorded. Changes in intracellular Ca^2+^ concentrations were measured and recorded every 5 s within the same region of a cell, and plotted for 5 min. The fluorescent signals in human U87 MG glioblastoma cells were quantified and statistically analyzed.

### 4.9. Bioinformatic Approach

An NF-κB-specific DNA-binding element (5′-GGGRNYYYCC-3′) in the 5′-promoter region of the *aqp4* gene was predicted using the PROMO system [48]. There are five NF-κB-specific DNA-binding elements that exist in the 5′-promoter region of the *aqp4* gene.

### 4.10. NF-κB Reporter Assay

Transcription activity of NF-κB for regulating *aqp4* gene expression was analyzed using a reporter assay as described previously [49]. Briefly, human U87 MG glioblastoma cells were transfected with NF-κB luciferase reporter plasmids and a FuGene transfection reagent (Promega). In addition, blank pUC18 plasmids were transfected into human U87 MG glioblastoma cells as the negative control. After transfection of these two types of plasmids, human glioblastoma cells were treated with 100 nM bradykinin. Following drug treatment, cell lysates were prepared to measure the luciferase activity using a dual luciferase assay system (Promega). The luminance signals were measured and quantified using a sensitive monochromator-based microplate reader (BMG Labtech, Ortenberg, Germany).

### 4.11. Reverse-Transcription (RT) and Quantitative Polymerase Chain Reaction (qPCR) Analyses

Expression of AQP4 mRNA was quantified using an RT-PCR and qPCR as described previously [50,51]. After drug treatment, total RNAs from U87 MG cells were prepared. The respective upstream and downstream primers of oligonucleotide sequences, designed and synthesized by MDBio (Taipei, Taiwan), were 5’-GAATCCTCTATCTGGTCACA-3’ and 5’-TGTTTGCTGGGCAGCTTTG-3’ for human AQP4 mRNA [52], 5’-CTGGAGCCAGCATGAATC-3’ and 5’-TCTTCTCTTCTCCACGGTCA-3’ for murine AQP4 mRNA [53], and 5’-GTGGGCCGCTCTAGGCACCAA-3’ and 5’-CTCTTTGATGTCACGCACGATTTC-3’ for β-actin mRNA [50]. These mRNAs were reverse-transcribed into their complementary (c)DNAs. The AQP4 and β-actin cDNAs were amplified with initial denaturation at 94 °C for 5 min, followed by 35 cycles (94 °C for 45 s, 55 °C for 45 s, and 72 °C for 90 s), a final extension step at 72 °C for 10 min, and a stopping step at 4 °C. PCR products were loaded onto a 1.8% agarose gel containing 0.1 μg/mL ethidium bromide and electrophoretically separated. DNA bands were visualized and photographed under ultraviolet-light exposure. RT-PCR analyses of AQP4 and β-actin mRNA were conducted for at least 3 independent determinations. Levels Intensities of the DNA bands in the agarose gel were quantified with the aid of a digital imaging system (UVtec). A qPCR analysis was carried out using iQSYBR Green Supermix (Bio-Rad, Hercules, CA, USA) and the MyiQ Single-Color Real-Time PCR Detection System (Bio-Rad) as described previously [50]. 

### 4.12. Distribution of AQP4 in Human Glioblastoma Cells

The distribution of AQP4 in human malignant glioblastoma cells was analyzed as described previously [54]. Briefly, AQP4 was recognized by a specific antibody and visualized using confocal microscopy. Following administration of bradykinin, human U87 MG cells were harvested, washed, and then fixed in a mixture of acetone and methanol (1:1). After rehydration, human glioblastoma cells were reacted with Triton X-100 (0.2%) at room temperature. A mouse mAb was generated against human AQP4 (Cell Signaling). Distribution of AQP4 in U87 MG cells was immunodetected overnight. After washing, cells were successively incubated with second antibodies and biotin-SP-conjugated goat anti-rabbit immunoglobulin G (IgG) at room temperature for 1 h. Following washing, human glioblastoma cells were reacted with a third antibody with Cy3-conjugated streptavidin at room temperature for 30 min. Nuclei of U87 MG cells were stained with 4′,6-diamidino-2-phenylindole. A confocal laser scanning microscope (Olympus, Tokyo, Japan) was used for sample observation. Illumination of the existence of the AQP4 protein was established by the appearance of hot spots in the cytoplasm (red signals). Nuclei were stained with blue signals. Images were acquired and quantified using FluoView software (Olympus).

### 4.13. Knockdown of BDKRB1

Expression of BDKRB1 in glioblastoma cells was knocked-down using an RNA interference (RNAi) technique as described previously [39]. The B1 bradykinin receptor small interfering (si)RNAs, purchased from Santa Cruz Biotechnology, were transfected into human U87 MG glioblastoma cells according to a transfection protocol provided by Santa Cruz Biotechnology. Briefly, after culturing human glioblastoma cells in antibiotic-free DMEM at 37 °C in a humidified atmosphere of 5% CO_2_ for 24 h, the BDKRB1 siRNA duplex solution, which was diluted in siRNA transfection medium (Santa Cruz Biotechnology), was added to U87 MG cells. After transfection for 24 h, the medium was replaced with normal DMEM, and human U87 MG glioblastoma cells were treated with bradykinin. Scrambled siRNA, purchased from Santa Cruz Biotechnology, was applied to human U87 MG glioblastoma cells as a negative standard.

### 4.14. Wound-Healing Assay

A wound-healing assay was carried out to determine the effects of bradykinin on the migration of human malignant U87 MG glioblastoma cells as described previously [40]. Briefly, human U87 MG and murine GL261 glioblastoma cells were grown in 12-well tissue culture plates at a density of 5 × 10^4^ cells/well. When glioblastoma cells had grown to confluence, the monolayers were scratched with a sterile 1-mL pipette tip. After washing with PBS, human and murine glioblastoma cells were treated with 100 nM bradykinin for 24 h. After bradykinin treatment, the wound area was observed and photographed under a light microscope (Nikon). In the scratched region, the area that was not occupied by migrated glioblastoma cells was calculated and statistically analyzed.

### 4.15. Matrigel-Based Invasion Assay

The effects of bradykinin on the migration of human and murine glioblastoma cells were further confirmed using a Matrigel-based invasion assay as described previously [55]. Costar transwell cell culture chamber inserts with an 8-μm pore size (Corning Costar, Cambridge, MA, USA) were soaked in 100 μL Matrigel matrix (BD Biosciences at 4 °C for 16 h and then incubated at 37 °C for another 2 h. Costar transwells were washed three times with DMEM and inserted in 12-well tissue cluster plates (Corning Costar). Human and murine glioblastoma cells (10^5^) suspended with bradykinin in 0.5 mL of rich medium were added to the inside of the transwells and cultured at 37 °C for 24 h in an atmosphere of 5% CO_2_. Culture medium (500 μL) containing 20% FBS was placed in the lower chambers. After treatment with bradykinin, glioblastoma cells on the upper surface of the transwells were fixed using methanol for 5 min and stained with Liu’s A. Then, fixed cells were photographed and counted under a microscope (Nikon). For each replicate, tumor cells in 10 randomly selected fields were determined, and counts were averaged.

### 4.16. Statistical Analysis

Each value represents the mean ± standard deviation (SD) for at least three independent determinations. Representative morphological and confocal images as well as immunoblots and DNA agarose gels are presented in this study. All of the signals for each assay were individually quantified and statistically analyzed. Statistical differences between the control and drug-treated groups were considered significant when the *p* value of Duncan’s multiple-range test was < 0.05. Statistical analysis between drug-treated groups was carried out using a two-way analysis of variance (ANOVA).

## 5. Conclusions

In conclusion, this study revealed upregulation of AQP4 mRNA and protein expression in human glioblastomas. Bradykinin specifically elevated levels of BDKRB1 in human glioblastoma cells and stimulated an influx of Ca^2+^. Sequentially, exposure to bradykinin augmented phosphorylation of MEK1 and ERK1/2. Accordingly, translocation and transactivation activities of NF-κB were enhanced by bradykinin. Bradykinin induced AQP4 mRNA and protein expressions. Migration and invasion of human glioblastoma cells were raised after exposure to bradykinin. Knocking-down BDKRB1 simultaneously inhibited AQP4 expression and migration and invasion by human brain tumor cells. Bradykinin-induced alterations in human glioblastoma cells were also confirmed in murine glioblastoma cells. As a result, the bradykinin-BDKRB1/2 axis contributes to regulation of AQP4 expression as well as migration and invasion of glioblastoma cells. The bradykinin-BDKRB1 axis may be a target for therapy of glioblastomas. In the future, orthotopic models of brain tumors will be created in order to support our in vitro findings.

## Figures and Tables

**Table 1:** Effects of bradykinin on survival, intracellular calcium concentrations, AQP4 mRNA expression, wound healing, and invasion of murine GL261 glioblastoma cells.

Cell Activities	Control	Bradykinin
Cell survival, cell numbers × 10^3^	46 ± 9	43 ± 10
[Ca^2+^]_i_, fluorescent intensities × 10^3^	0.8 ± 0.2	16.2 ± 3.5 *
AQP4 mRNA, quantity of relative expression	1 ± 0.2	3.2 ± 0.7 *
Wound healing, % of area	57 ± 11	23 ± 5 *
Cell invasion, cell numbers	49 ± 9	578 ± 99 *

Murine GL261 glioma cells were treated with bradykinin for different intervals. Cell survival was determined using a trypan blue exclusion assay. Levels of intracellular calcium [Ca^2+^]_i_ influx were analyzed using confocal microscopy. AQP4 mRNA was quantified with a real-time PCR. Migration of GL261 cells was examined using wound healing and matrigel-based migration assays. An asterisk (*) indicate that a value significantly (*p* < 0.05) differed from the respective control.

## Supplementary Materials

The following are available online at https://www.mdpi.com/2072-6694/12/3/667/s1, Supplementary figures for whole blot.
