# Supplementary figures and images for: The Bradykinin-BDKRB1 Axis Regulates Aquaporin 4 Gene Expression and Consequential Migration and Invasion of Malignant Glioblastoma Cells via a Ca2+-MEK1-ERK1/2-NF-κB Mechanism
Source: Cancers (Basel). 2020 Mar 13;12(3):667. doi: 10.3390/cancers12030667 (PMC7139930; doi:10.3390/cancers12030667)

Fig. 2E

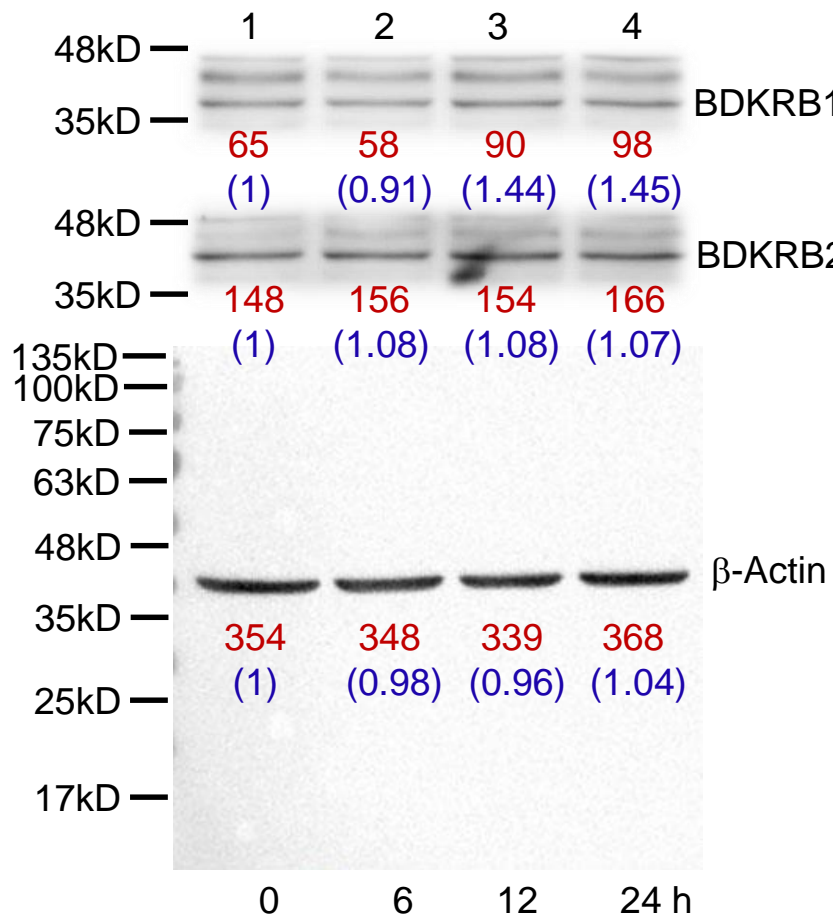

Fig. 3A

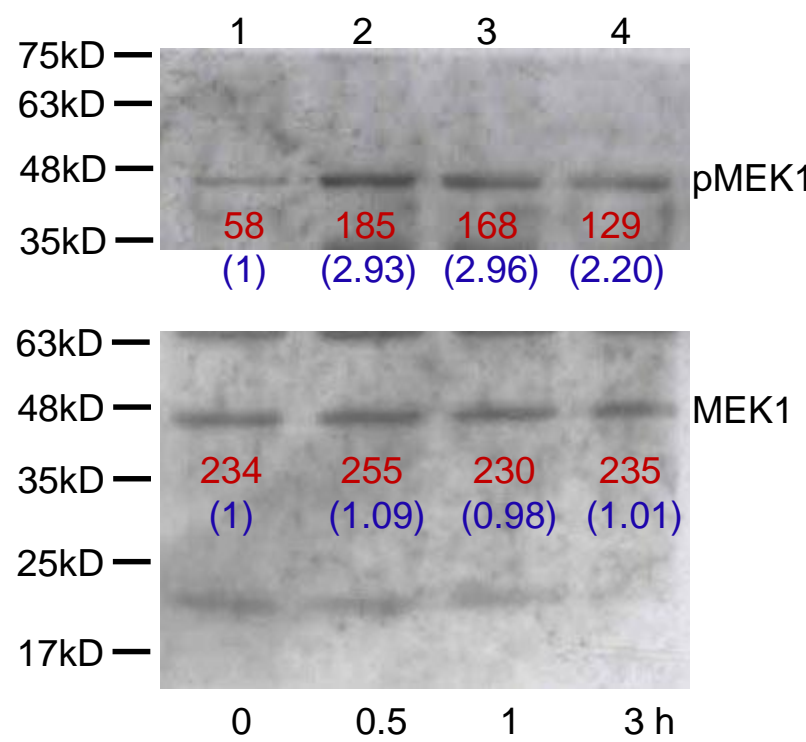

Fig. 3C

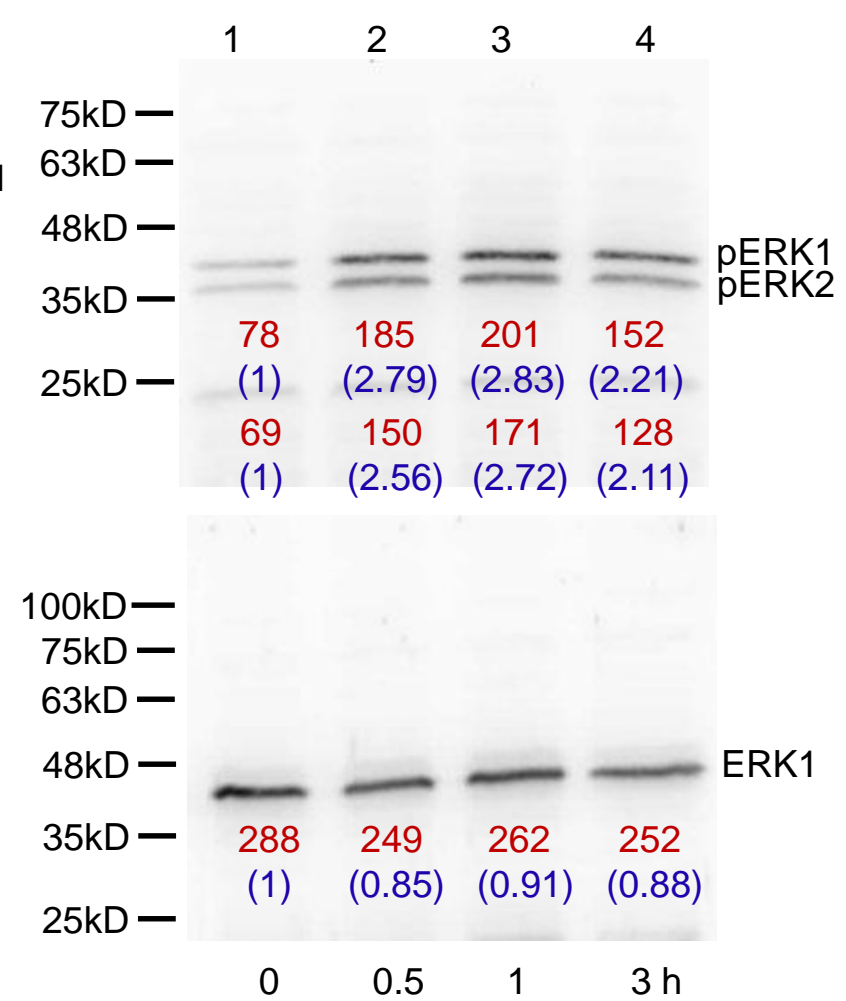

Fig. 4A

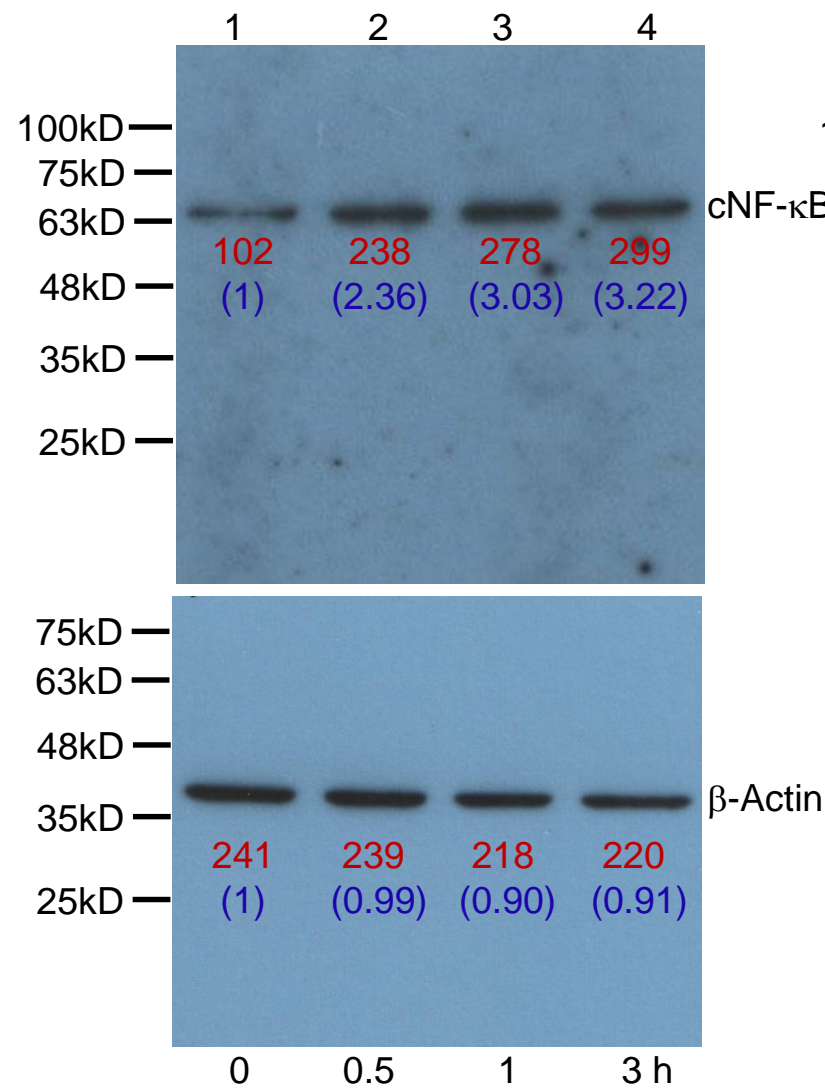

Fig. 4C

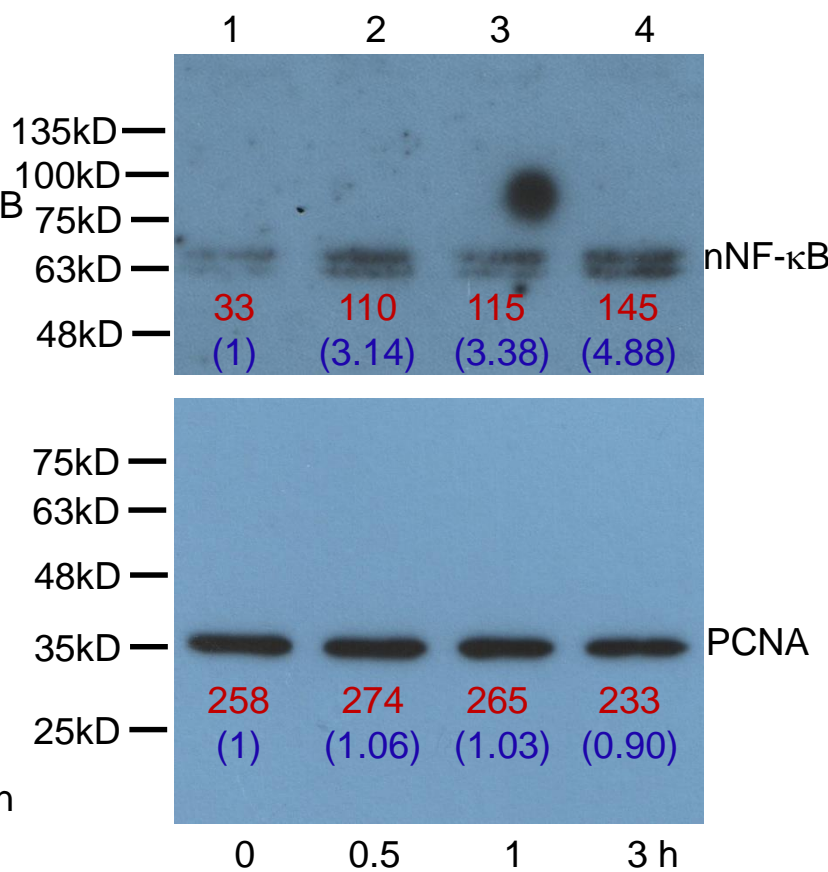

Fig. 5F

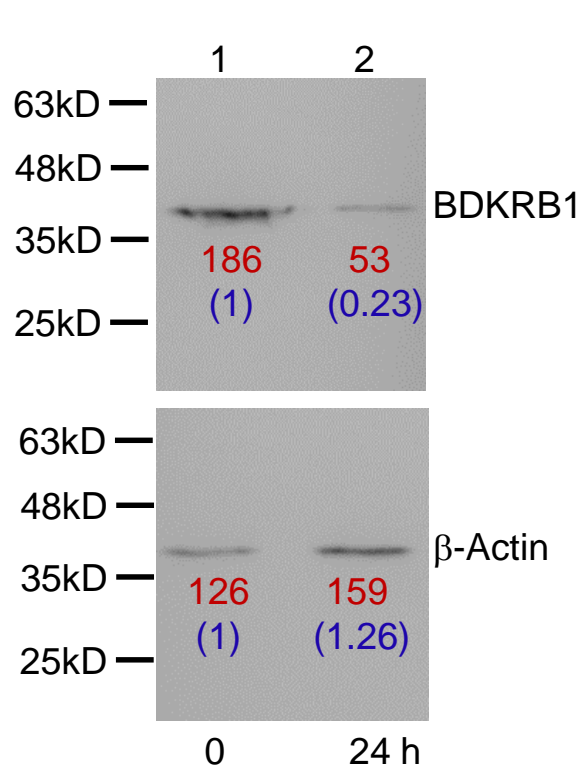

Supplement: Supplementary file 1 [file cancers-12-00667-s001.pdf]
